# Anti-Inflammatory, Anti-Apoptotic, and Antioxidant Roles of Honey, Royal Jelly, and Propolis in Suppressing Nephrotoxicity Induced by Doxorubicin in Male Albino Rats

**DOI:** 10.3390/antiox11051029

**Published:** 2022-05-23

**Authors:** Hanaa K. Mohamed, Maysa A. Mobasher, Rasha A. Ebiya, Marwa T. Hassen, Howaida M. Hagag, Radwa El-Sayed, Shaimaa Abdel-Ghany, Manal M. Said, Nabil S. Awad

**Affiliations:** 1 Department of Zoology, Faculty of Women for Arts, Science and Education, Ain Shams University, Cairo 11757, Egypt; hanaa.khairy@women.asu.edu.eg (H.K.M.); rasha.ebiya@women.asu.edu.eg (R.A.E.); marwa.hassen@women.asu.edu.eg (M.T.H.); radwa.ahmed@women.asu.edu.eg (R.E.-S.); 2 Department of Pathology, Biochemistry Division, College of Medicine, Jouf University, Sakaka 41412, Saudi Arabia; 3 Department of Pathology, Faculty of Medicine, Al-Azhar University, Nasr City, Cairo 11884, Egypt; howaida.m@tu.edu.sa; 4 Department of Clinical Laboratory Sciences, College of Applied Medical Sciences, Taif University, Taif 21944, Saudi Arabia; 5 College of Biotechnology, Misr University for Science and Technology, Giza 12563, Egypt; shaimaa.ibraheem@must.edu.eg (S.A.-G.); manalfaris@yahoo.com (M.M.S.); nabil.faris@must.edu.eg (N.S.A.); 6 Department of Genetics, Faculty of Agriculture and Natural Resources, Aswan University, Aswan 81528, Egypt

**Keywords:** doxorubicin, nephrotoxicity, PARP-1, Bcl-2, TNF-α, honey, royal jelly, propolis

## Abstract

Nephrotoxicity is one of the limiting factors for using doxorubicin (DOX). Honey, propolis, and royal jelly were evaluated for their ability to protect against nephrotoxicity caused by DOX. Forty-two adult albino rats were divided into control groups. The DOX group was injected i.p. with a weekly dose of 3 mg/kg of DOX for six weeks. The DOX plus honey treated group was injected with DOX and on the next day, received 500 mg/kg/day of honey orally for 21 days. The DOX plus royal jelly treated group was injected with DOX and on the following day, received 100 mg/kg/day of royal jelly orally for 21 days. The DOX plus propolis treated group received DOX and on the following day, was treated orally with 50 mg/kg/day of propolis for 21 days. The DOX plus combined treatment group received DOX and on the following day, was treated with a mix of honey, royal jelly, and propolis orally for 21 days. Results confirmed that DOX raised creatinine, urea, MDA, and TNF-α while decreasing GPX and SOD. Damages and elevated caspase-3 expression were discovered during renal tissue’s histopathological and immunohistochemical studies. Combined treatment with honey, royal jelly, and propolis improved biochemical, histological, and immunohistochemical studies in the renal tissue. qRT-PCR revealed increased expression of poly (ADP-Ribose) polymerase-1 (PARP-1) and a decline of Bcl-2 in the DOX group. However, combined treatment induced a significant decrease in the PARP-1 gene and increased Bcl-2 expression levels. In addition, the combined treatment led to significant improvement in the expression of both PARP-1 and Bcl-2 genes. In conclusion, the combined treatment effectively inhibited nephrotoxicity induced by DOX.

## 1. Introduction

Doxorubicin (DOX), an anthracycline antibiotic, has been applied as an effective anti-cancer therapy since 1969 [1,2]. Despite its potent anti-cancer properties, the clinical usefulness of DOX in cancer chemotherapy is limited by its severe consequences on non-targeted organs, including the kidneys, liver, and brain [3]. DOX was reported to raise the permeability of glomerular capillaries and atrophy of the glomeruli in rat kidneys [4]. After DOX accumulation, severe glomerular damage was produced due to oxidative stress [5]. Lipid peroxidation and diminished antioxidant enzyme activity are common mediators promoting nephrotic syndrome [6]. Inflammation plays an influential role in renal damage exhibited by DOX through cytokines and other cytotoxic factors [7]. Protein excretion due to renal failure is primarily associated with damage to the filtration barrier [7]. This filtration barrier deterioration is caused by DOX [8]. Several researchers reported that DOX caused alterations in the kidney function parameters. Manal et al. (2019) published that DOX caused significant increases in creatinine and urea levels [9]. DOX causes oxidative stress in kidney tissues characterized by low antioxidant enzyme activity and enhanced malondialdehyde activity [10]. DOX-induced nephrotoxicity causes increased vascular porousness and glomerular contraction. It is marked by increased kidney functions and raised lactate dehydrogenase activity, along with decreased renal Ca2+- ATPase, Mg2+-ATPase, and Na+, K+-ATPase rates [11,12]. Natural antioxidants reduce harmful side effects and improve the antitumor activity of anti-cancer medications [13,14,15]. Different studies have demonstrated that a diet high in honey and bee products (propolis and royal jelly) provides significant health benefits against various diseases due to their antioxidant properties [16,17].

Honey is a naturally occurring food that is produced by bees. It is known worldwide for its great nutrient ingredients beneficial to humans. Major sugars and vitamins, phenolic acids, minerals, and phytochemicals are found in honey. Egyptians, Greeks, Romans, and Chinese have all utilized honey to treat wounds and intestinal disorders, including gastric ulcers. It has also been used to treat earache, coughs, and sore throats [18,19]. Honey has lately been used for its anti-inflammatory, antimicrobial, and antioxidant activities and for enhancing the immune response [20]. Honey’s biological activity can be related to its polyphenolic content, linked to its antioxidant and anti-inflammatory properties, and its cardiovascular, anti-proliferative, and antibacterial benefit [21,22].

Royal jelly is made from the mandibular glands and hypopharyngeal of worker honeybees as a milky secretion [23]. It contains royalactin proteins, monosaccharides, lipids, fatty acids, minerals, free amino acids, and vitamins. Antitumor, anti-inflammatory, antioxidant, hypoglycemic, and hypo-cholesterolemic actions are considered biological benefits of royal jelly [24,25].

Propolis, another defense product from the bee, is collected from different plant sources. Additionally, propolis has several biological properties, including antioxidant and free radical scavenger action, antimicrobial activity against a broad spectrum of pathogens, anti-malignant action, anti-inflammatory activities, and immune-boosting properties by promoting numerous pro-inflammatory cytokines [19]. Propolis was reported to enhance the protective effect on kidney failure induced by paracetamol, carbon tetrachloride, and Doxorubicin [26,27]. The current study aims to assess the preventive roles of natural honey, royal jelly, and propolis administration, both alone and combined, on Dox-induced renal toxicity. The biochemical, histopathological, immunohistochemical, and gene expression modifications in albino rats were examined.

## 2. Materials and Methods

### 2.1. Chemicals and Natural Products

Doxorubicin (DOX) hydrochloride, honey, royal jelly, and propolis were obtained from Sigma chemical company (St. Louis, MO, USA).

### 2.2. Experimental Animals

The protocol of the Institutes of Health (NIH) and the Commission for Control and Supervision of Experiments on Animals (CPCSEA) (registration number: 13/165) were performed in our experimental animal study. Our research was conducted in the biology lab of Ain Shams University’s Department of Zoology, Faculty of Women for Arts, Science, and Education. The rats used in this investigation were 42 adult male albino rats weighing (150–160 g). Rats were kept in a properly ventilated room and exposed to natural light (12:12 h light-dark cycle). In the laboratory, the animals were housed in metabolic labeled cages at a constant temperature of 25 °C. They had unrestricted access to conventional dry pellet food and water. They were acclimated for a week before the experiment started.

### 2.3. Induction of Nephrotoxicity

Doxorubicin (DOX) hydrochloride 10 mg vial (Pharmacia Italia, Milano, Italy) was injected intraperitoneally in six equal doses (i.p., 3 mg/kg b.w.) for six weeks (one dosage each week for a total dose was18 mg/kg b.w.) as previously described [28]. All other chemicals and reagents used were of analytical grade.

### 2.4. Experimental Design

Forty-two male albino rats were divided into six groups of seven animals each. The control group (NG) was given saline; the Doxorubicin group (DOX-G) was administrated with a single dose of 3 mg/kg/week i.p. of DOX for six weeks for kidney toxicity induction; the Dox plus honey treated group (DOX-H) was introduced with DOX (single dosage of 3 mg/kg/week i.p. for six weeks) and on the next day receiving 500 mg/kg/day of honey orally for 21 days; the Dox plus royal jelly treated group (DOX-R) treated with DOX (only one dose 3 mg/kg/week i.p. for six weeks) and on the next day treated orally with 100 mg/kg/day royal jelly for 21 days; the Dox plus propolis treated group (DOXP) received DOX (single dose 3 mg/kg/week i.p.) and on the next day treated orally with 50 mg/kg/day propolis for 21 days; the Dox plus honey, royal jelly, and propolis combined group (DOXHRP) received DOX (15 mg/kg, i.p.) and on the subsequent day treated with 500 mg/kg/day of honey, 100 mg/kg/day Royal jelly, and 50 mg/kg/day propolis orally for 21 days.

At the end of the experiment, non-heparinized capillary tubes were used to collect blood samples from the retro-orbital plexus. To analyze kidney functions, serum specimens were produced after centrifuging for 20 min and collecting the supernatants. The kidneys were promptly divided and cleaned with saline after decapitating the rats. One portion of the kidney was homogenized in phosphate-buffered saline and centrifuged to prepare 25% *w*/*v* tissue homogenates. After that, the supernatants were collected and kept at −80 °C until analysis. For histopathology and immunohistochemistry examination, another kidney section was rinsed with saline and then put in 10% formal saline. The third part was kept at −80 °C in trizol for gene expression analyses.

### 2.5. Biochemical Determinations

Renal Biomarkers: Biodiagnostic Co. Egypt kit was used as a colorimetric method to assay urea, as previously described [29]. Creatinine was measured using a Kit obtained from Diamond Diagnostics Co., Cairo, Egypt, according to [30].

Antioxidants and oxidative markers: Biodiagnostic Co., Egypt kits were used in colorimetric methods to determine renal malondialdehyde (MDA), superoxidase dismutase (SOD), and glutathione peroxidase (GPX) according to [31,32,33,34], respectively.

Tumor necrosis factor-α (TNF-α) and B-Cell Leukemia/Lymphoma 2 (Bcl2): Tumor necrosis factor-α (TNF-α) content was evaluated by ELISA technique using a TNF-α assay kit acquired from Assay Pro., Co., Charles city, IA, USA, following the procedure described by [35]. B-Cell Leukemia/Lymphoma 2 (Bcl2) was measured using the ELISA technique following the manufacturer’s instructions for ELISA KIT of rat Bcl2 procured from Cloud-Clone Corp. (CCC, Houston, TX, USA).

### 2.6. Cell Examination of Kidney Tissues

Dissected renal tissue samples were rinsed in normal saline and fixed in 10% saline for 72 h. After that, the specimens were cut and dried in alcohol, then cleared in xylene, filtered in wax, and finally blocked out into Paraplast tissue embedding media. A rotatory microtome was used to cut 5 µm thick sections of each sample. According to the previously reported methodology, the sections were stained with hematoxylin and eosin (H&E) stain [36]. Slides were examined under a microscope at 400× magnification.

### 2.7. Immuno-Histochemical Study (Caspase-3)

Caspase 3 antibodies were stained immunohistochemically at dilution (1:50) and on 4-μm, paraffin-embedded sections. Antigen retrieval in all samples was accomplished by heating the plates for 30 min in a solution of EDTA (1-mmol/L, pH 8.0), followed by endogenous biotin inhibition. Staining with an automated immune Stainer (DAKO) was performed, then detected with a streptavidin-biotin detection system. Additionally, positive and negative control sections were applied.

### 2.8. Gene Expression Analysis

The changes in mRNA levels of PARP1 and Bcl2 genes were assessed using quantitative real-time PCR [37,38]. An internal control GAPDH was used as a standard for the RT- PCR analysis. Trizol reagent (Invitrogen Life Technologies, Carlsbad, CA, USA) was used to extract the total RNA of kidney tissue following the supported manufacture. The spectrophotometer was used to investigate the purity and concentration of total RNA. The cDNA synthesis kit (Takara, Kyoto, Japan) was used for cDNA synthesizing according to the manufacturer’s instructions. Quantitative RT-PCR was conducted using the SYBR Green mix kit (Applied Biosystems, Foster City, CA, USA) applying Mini TM thermocycler (Bio-Rad Laboratories Inc., New York, CA, USA). In a final volume of 25 µL, 2 µL cDNA was added to 12.5 µL 2× SYBR Green mix, 1 µL of each forward and reverse primers, and 8.5 µL of deionized distal water. The primers sequences were designed using primer design software Primer 3 version 4.1.0 online at https://primer3.ut.ee/ (accessed on 31 March 2022): Bcl-2 (F) 5′-TTTGATTTCTCCTGGCTGTCT-3′ and (R) 5′-CTGATTTGACCATTTGCCTG-3′; PARP-1 (F) 5′-TCTCCAATCGCT TCTACACCCT-3′ and (R) 5′-TACTGCTGTCATCAGACCCACC-3′; GAPDH (F) 5-GCAAGTTC GCAAGTTCAACGGCACAGTCAAG-3 and (R) 5-GTACTCAGCACCAGCATCACC-3(The PCR reactions followed programs of first 95 °C for 10 min, then 40 cycles consisting of 94 °C for 15 s, and 60 °C for 1 min, finally melting curve analysis was used to specify the amplification. gene accession number Bcl_2, PARP-1, NM_013063.2 and NM_016993.2) The 2∆∆CT method was used to analyze the obtained data. The mRNA levels results were generalized against the amount of housekeeping gene GAPDH Results were presented as fold change relative to the negative control (RFC) [39].

### 2.9. Statistical Analysis

The SPSS program version 25 (IBM Corp., Armonk, NY, USA) was used to analyze the obtained data statistically. The mean and standard error were used to summarize our data. For comparisons between groups, variance analysis (ANOVA) was used with a multiple comparisons post hoc test for every two groups.

## 3. Results

### 3.1. Renal Biomarkers

**Figure 1:**
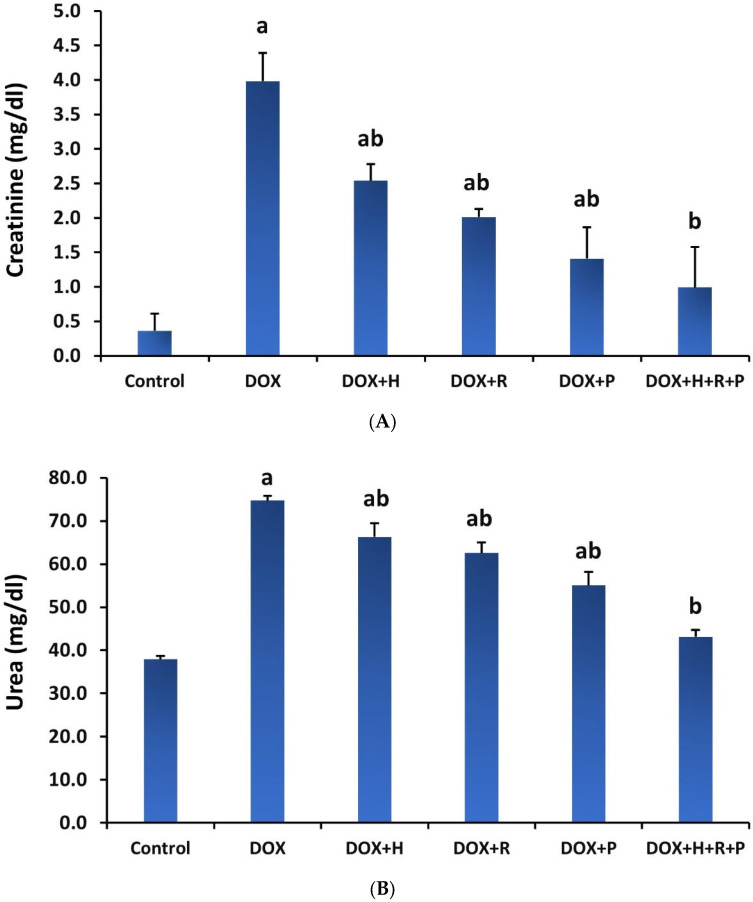
Effect of honey (H), royal jelly (R), and propolis (P) on the renal functions in rats treated with Doxorubicin, (**A**) creatinine, and (**B**) urea. Values are shown as mean ± SE. Different lowercase letters indicate significant differences compared to the corresponding value in the control group at *p* < 0.05.

The findings in Figure 1A,B show that serum creatinine and urea levels in the DOX group were significantly higher (*p* ≤ 0.05) than in the control group. However, compared to the DOX group, treatment of H, R, or P alone or a mixture of H, R, and P led to a substantial reduction (*p* ≤ 0.05) in the serum urea and creatinine levels.

### 3.2. Antioxidants Status

**Figure 2:**
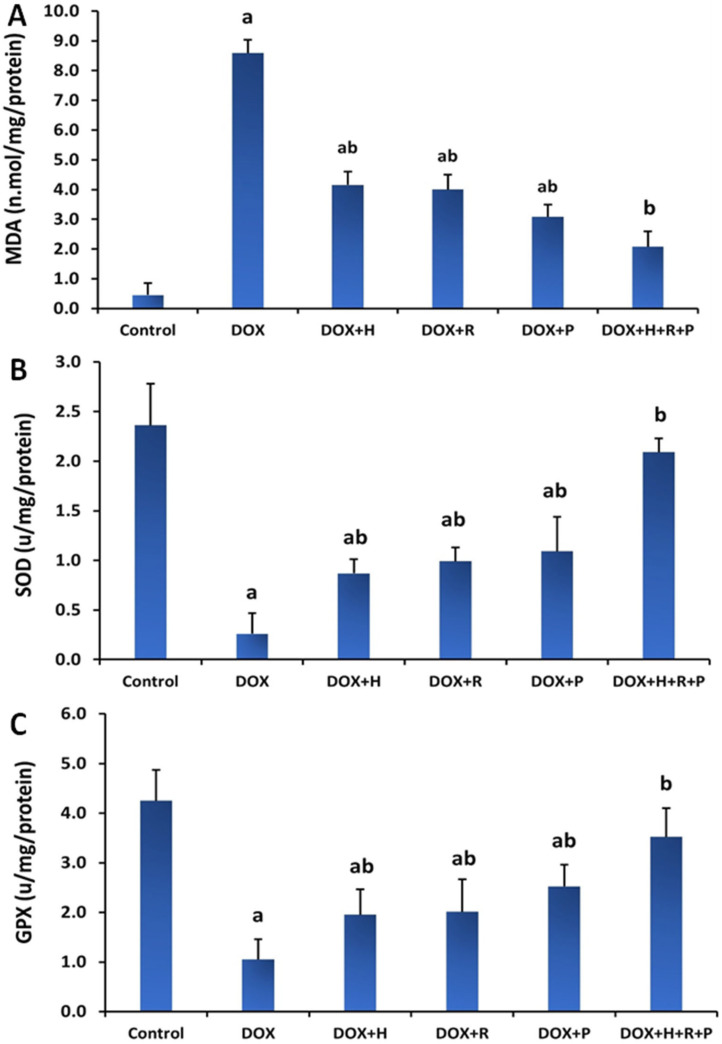
Effect of honey, royal jelly, and propolis on the oxidative marker and antioxidant enzyme activity in renal tissues of rats treated with Doxorubicin, (**A**) MDA, (**B**) SOD, and (**C**) GPX. Values are shown as mean ± SE. Statistically, Different lowercase letters indicate significant differences compared to the corresponding value in the control group at *p* < 0.05.

The effects of H, R, and P, alone or combined of all H, R, and P on MDA and enzymatic antioxidants in DOX-treated rats are shown in Figure 2A,B. The DOX group had a significant rise in renal MDA content (*p* ≤ 0.05) in contrast to the control group. Treatment of the DOX group with H, R, and P or all of H, R, and P together, on the other hand, reversed this rise as evidenced by a significant decline (*p* ≤ 0.05) in MDA concentration when compared to the DOX group. Compared to controls, the DOX group had significantly lower SOD and GPX. As the DOX group was treated with H, R, and P, alone or combined of all H, R, and P together, the level of SOD and GPX in the kidney homogenate increased (*p* ≤ 0.05) relative to the DOX group.

### 3.3. Inflammatory Markers

**Figure 3:**
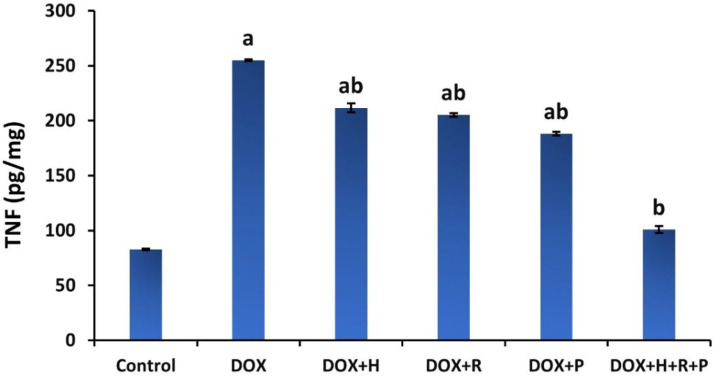
Effect of honey, royal jelly, and propolis on TNF-α of rats treated with Doxorubicin. Values are shown as mean ± SE. Statistically. Different lowercase letters indicate significant differences compared to the corresponding value in the control group at *p* < 0.05.

Results in Figure 3 demonstrate the treatment with H, R, and P, alone or combined of H, R, and P together on renal TNF-α in the DOX group. The DOX group showed a significant increase (*p* ≤ 0.05) in TNF-α levels compared to the control group. In contrast, when the DOX group was treated with H, R, and P, alone or combined with H, R, and P, the renal levels of TNF-α were markedly decreased (*p* ≤ 0.05) compared to the untreated DOX group.

### 3.4. Histopathological Analysis: Haematoxylin and Eosin

**Figure 4:**
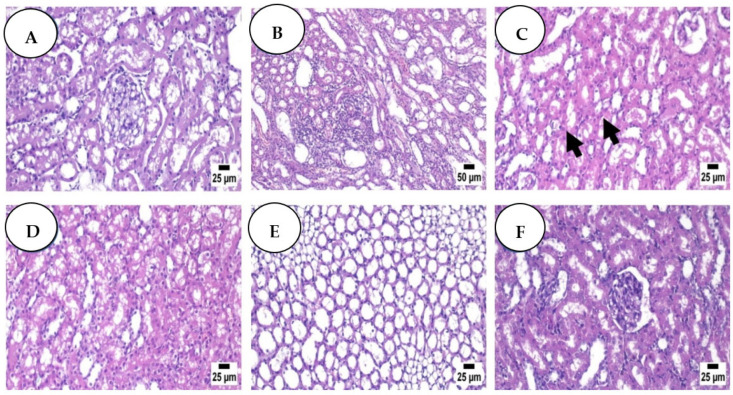
Control group (**A**) high power view shows average glomeruli with average Bowman’s spaces, average proximal tubules with preserved brush borders (black arrow), average distal tubules, and average interstitium (H&E); Dox group (**B**) showing focal cystic dilation of renal tubules with focal interstitial nephritis (H&E); Dox + H group (**C**) showing apparently normal renal cortex with few tubules containing renal cast (arrow) (H&E); (**D**) (Dox + R), showing some degenerating renal tubules (H&E); Dox + P group (**E**) showing apparently normal renal medulla (H&E); Dox + H + R + P group (**F**) showing apparently normal renal cortex (H&E).

The control group’s kidney sections (Figure 4A) revealed an average renal capsule, average glomeruli with average Bowman’s spaces, average proximal tubules with preserved brush borders, average distal tubules, and an average renal medulla with average collecting tubules, average epithelial lining, and average interstitium. In the DOX group, the kidney showed average renal capsule, atrophied glomeruli with enlarged Bowman’s gaps, proximal tubules with apoptotic epithelial lining, partial loss of brush borders, and intra-tubular debris notably dilated congested interstitial blood vessels with areas of hemorrhage. The renal medulla demonstrated collecting tubules with apoptotic epithelial lining and congested peri-tubular capillaries (Figure 4B). Regarding the renal medulla of the Dox + H group (Figure 4C), no histopathological changes were detected except for a few sections that exhibited marked congestion. Similarly, the renal medulla of the Dox + R group (Figure 4D) was apparently normal except for some sections that showed renal tubular degeneration and necrosis. Figure 4E (Dox + P) and Figure 4F (DOX + H + R + P) show an apparently normal renal cortex with congestion in some instances while exhibiting an apparently normal renal medulla.

### 3.5. Immuno-Histochemical Studies: Caspase 3

**Figure 5:**
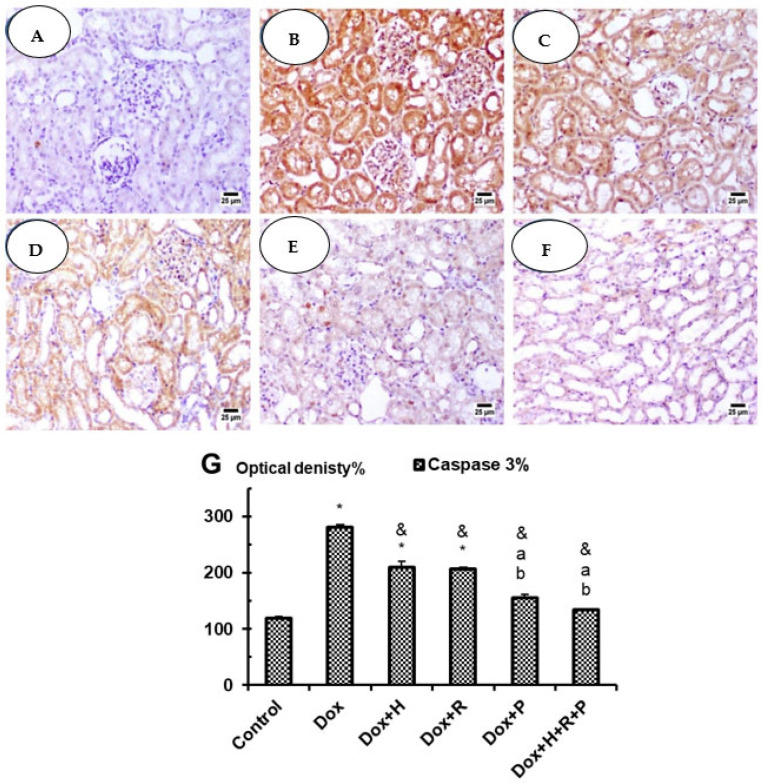
Control group (**A**) high power view showing negative expression of caspase 3 (immunostaining); Dox group (**B**) showing higher expression of caspase 3 (immunostaining); Dox + H group (**C**) and Dox + R group (**D**) showing moderate expression of caspase 3 (immunostaining); Dox + P group (**E**) and Dox + H + R + P group (**F**) show weak expression of caspase 3 (immunostaining). (**G**) Optical density% for Caspase 3 in experimental groups. Results are expressed as mean ± S.E.M. and analyzed using one-way ANOVA followed by Bonferroni’s test for multiple comparisons. * *p* ≤ 0.05 versus control group. ^&^ *p* ≤ 0.05 versus Dox group. ^a^ *p ≤ 0.05* versus Dox + H group. ^b^ *p* ≤ 0.05 versus Dox + R group. *n* = 5.

In the control group, the kidneys showed negative reactivity (0) for caspase-3 in glomeruli, negative cytoplasmic reactivity (0) in proximal tubules, and negative reactivity (0) in collecting tubules (Figure 5A); however, in the DOX Group, the kidneys showed an average renal capsule, atrophied glomeruli with widened Bowman’s spaces, proximal tubules with apoptotic epithelial lining with high expression of caspase 3% when compared with the control group at *p* ≤ 0.05 (Figure 5G), partial loss of brush borders and intra-tubular debris, and markedly dilated congested interstitial blood vessels with areas of hemorrhage. The renal medulla showed collecting tubules with apoptotic epithelial lining and congested peri-tubular capillaries (Figure 5B). In particular Dox + H and Dox + R groups, the kidneys showed moderate cytoplasmic reactivity (++) for caspase-3 in glomeruli, moderate (++) in proximal tubules, and moderate reactivity (++) in collecting tubules (Figure 5C,D) with a significant decrease in optical density % of Caspase 3 in comparison with Dox group at *p* ≤ 0.05 (Figure 5G). On the other hand, kidneys in both Dox + P and Dox + H + R + P groups showed improvement in weak cytoplasmic reactivity (+) for caspase-3 (Figure 5E,F) with significant inhibition in optical density % of Caspase 3 in comparison with Dox group as well as Dox + H and Dox + R groups at *p* ≤ 0.05 (Figure 5G).

### 3.6. Gene Expression Analysis

**Figure 6:**
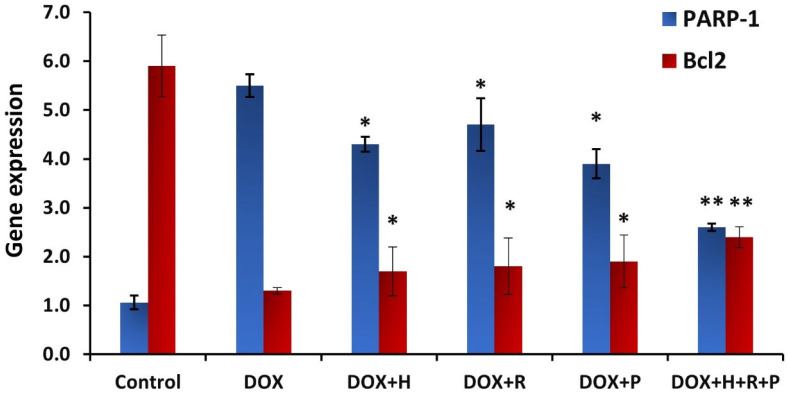
Histogram for poly (ADP-ribose) polymerase 1 (PARP-1) and Bcl2 genes expression in response to treatment with honey (H), propolis (P), and royal jelly (R) mix (H + P + R) on rats treated with Doxorubicin. The groups are negative control; Dox treated group (Dox); the Dox +hony treated group (Dox + H); the Dox + royal jelly treated group (Dox + R); the Dox +propolis treated group (Dox + P); the Dox + mix of honey, royal jelly, and propolis treated group (Dox + H + P + R). * mean significant *p*-value less than 0.05. ** mean significant *p*-value less than 0.01.

The present study detects changes in the mRNA expression levels of two genes as molecular biomarkers using real-time PCR. The inflammatory impact and anti-apoptotic effect of DOX and treatment with honey, royal jelly, and propolis were detected in kidney tissues. A statistically significant (*p* ≤ 0.05) increase in PARP-1 gene expression was determined after DOX injection compared to the control group (5.5, 1.06, respectively). The protective effect of honey, propolis, and royal jelly treatment was detected in the significant downregulation (*p* ≤ 0.05) of the PARP-1 gene expression level, whereas highly significant (*p* ≤ 0.01) downregulation was demonstrated after combined treatment of honey, royal jelly, and propolis as compared to the DOX group (4.3, 4.7, 3.9, 2.6, 5.5, respectively). The apoptotic molecular biomarker Bcl2 showed significant downregulation of Bcl2 gene expression after treatment with DOX (1.3) compared to the control group (5.9). Treatment with honey, propolis, and royal jelly after nephrotoxicity induction with DOX resulted in a significant (*p* ≤ 0.05) increase in Bcl2 mRNA expression and was highly significant with a combined honey, royal jelly, and propolis treatment as compared with the DOX group (1.7, 1.8, 1.9, 2.4, 1.3, respectively) (Figure 6).

## 4. Discussion

**Figure 7:**
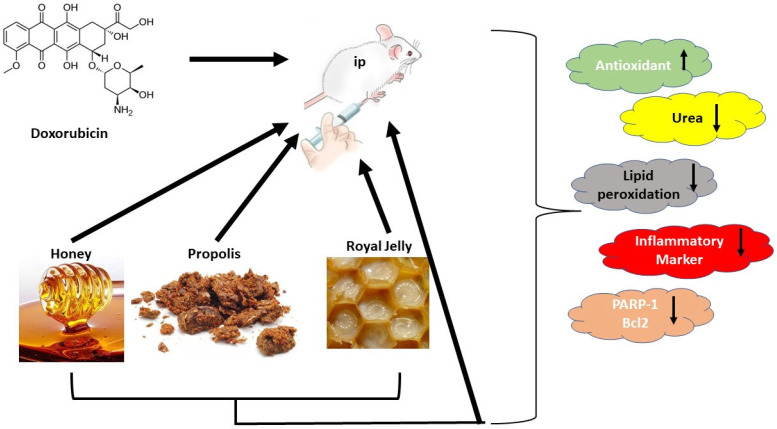
Schematic diagram showing the experimental design and relevant results. 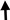 Upregulation of the parameter. 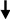 Downlegulation of the parameter.

Our study revealed a decline in the glomerular filtration level and a considerable increase in blood creatinine and urea after DOX administration. These findings were consistent with data previously published by [8,40]. They stated that chemotherapy causes acute renal failure with severe renal tubular impairments. The mechanism of DOX-inducing renal injury is through inflammation, which stimulates ROS production and apoptosis, with a decrease in antioxidant enzymes in the kidneys [41]. The most sensitive markers of nephrotoxicity are serum urea and creatinine [42]. The elevated creatinine level in the DOX group is related to DOX toxicity disrupting kidney function, which profoundly affects total body metabolism (Figure 7). Our investigation parallels the earlier analyses [43].

In contrast, the treatment with H, R, and P, or all of them as a mixture, improved abnormalities in renal parameters (serum creatinine and urea) caused by DOX. Previously, the utilization of honey was revealed to protect against cisplatin-induced kidney toxicity via the suppression of inflammation [44]. These findings established the protecting role of honey against DOX-induced kidney toxicity in rats. These results agree with the results found by Omotayo et al. (2012), Waykar et al. (2018), and Alhumaydhi (2020), who indicated that royal jelly and honey have preventive properties on renal dysfunctions [22,45,46]. Honey and royal jelly are both beneficial foods with high antioxidant capacity. They have hepato-protective, hypoglycemic, reproductive, and antihypertensive benefits. Several investigations declared the protective effect of honey in kidney functions against many drugs [47]. Another study reported the capability of honey to preclude hepato-nephrotoxicity-induced rats treated with cadmium. Additionally, the nephroprotective effect of propolis was evaluated by Baykara et al. by improving renal oxidation and decreasing serum creatinine urea levels which are similar to our data [48].

Promsan et al. studied pretreatment with one of the main constituents of propolis flavonoids, pinocembrin (5,7-dihydroxyflavone), which enhanced renal function and diminished apoptotic and oxidative stress markers [49]. These conclusions determined the protective effect of pinocembrin against nephrotoxicity because of its antioxidant and anti-apoptotic roles. It regulates the antioxidant enzymes and attenuates the rise in oxidative stress through Nrf2/HO-1 and NQO1 pathways [50]. An increase of MDA, an indicator of lipid peroxidation, is directly associated with free radical impairment to the glomerular basement membrane. The dismutation of O_2_ to H_2_O_2_ and molecular oxygen is catalyzed by the SOD enzyme, while GPX catalyzes the degradation of H_2_O_2_ to O_2_ and H_2_O. The reduction of SOD and GPX activities and increment of MDA content were revealed after DOX injection, resulting in diminished kidney ability to scavenge toxic H_2_O_2_ and lipid peroxides. These conclusions agree with El-Sheikh et al., who discussed the mechanism by which DOX-induced nephrotoxicity and cytotoxicity, evidenced by the breakdown of cell membranes and cellular components, is accelerated by oxidative stress generated by excess reactive oxygen species (ROS) [51]. ROS activity changes specific intracellular components, including proteins, lipids, and nuclear DNA [52].

The treatment with H, R, and P, separated or mixed, was proven to lower MDA levels and alter SOD and GPX levels. These results are consistent with earlier research that indicated an increase in antioxidant levels in honey use; this effect might be accompanied by the honey composition, such as many nutrients and antioxidants [53,54]. Additionally, these findings suggest a potent protective effect against oxidative stress, resulting in honey administration. Moreover, royal jelly’s high antioxidant capacity facilitates scavenging free radicals, lowering the nitric oxide level and subsequently reducing lipid oxidation and inhibiting protein oxidation, as reflected through the decline in renal function parameters. Additionally, honey and royal jelly serve an effective role in developing normal cellular immunity [55]. Propolis, a strong antioxidant rich in flavonoids, can scavenge free radicals and therefore protect the cell membrane against lipid peroxidation. Caffeic acid phenethyl ester (CAPE) is one of the main components of propolis, which can block ROS production in several systems [56]. Additionally, propolis induced upregulation of Nrf2 expression, the main intracellular transcription factor. It is released under oxidative stress from its repressor (Keap1) and thus restores antioxidant enzyme function. The released Nrf2 binds to the antioxidant response element (ARE) in the gene promoter of cytoprotective genes, stimulating their expression. Subsequently, to remove the effect of cytotoxic oxidants, the expression of free radical-scavenging enzymes occurred [57,58]. DOX administration produced a significant increase in TNF-α levels, a pro-inflammatory cytokine created by glomerular and tubular cells and outside injected inflammatory cells, and acts via mitogen-activated protein kinases (MAPKs) and nuclear factor kappa B (NF-κB) signaling pathways [59]. The initiation of these paths upregulates the expression of some inflammatory cytokines, such as TNF-α [60]. Al-Saedi et al. DOX-produced superoxide anion was found responsible for TNF-induced nuclear factor (NF) stimulation [61] and TNF upregulation [62].

After the DOX group was treated with H, R, and P, or a mixture of them, there was a noticeable increase in TNF-α levels. These findings were in accordance with Thi Lan Nguyen et al., who found that the anti-inflammatory activity of honey is due to its phenolic mixes and other minor constituents [63]. Ahmad et al. and Kassim et al. detected that quercetin, chrysin, ellagic acid, and ferulic acid hesperetin in honey are protective supplements for different inflammatory diseases [64,65]. Royal jelly treatment controlled the alterations of measured pro-inflammatory cytokine. Several reports documented the beneficial impact of royal jelly and its ingredients on anti-inflammatory activity in different experimental models. Moreover, one of the major lipid constituents in royal jelly is 10-hydroxy-2-decenoic acid, which was said to exert anti-inflammatory consequences in colon cancer cells passing through inhibiting NF-κB, which further inhibited the release of TNF-α. CAPE, the main constituent in propolis, may be responsible for propolis’s anti-inflammatory effects by lowering the inflammatory cytokines in the inflammatory cells [66].

The histological examination in the present study revealed glomerular congestion, tubular degeneration, vacuolization, necrosis, hyaline cast, brush border loss of proximal cells, and epithelial cell detachment in the DOX group. These alterations were linked to the failure of renal functions, such as elevated creatinine and urea levels. These results were attributed to DOX, which was absorbed by the kidney’s tubular cells, especially in proximal tubules. Additionally, this result could be associated with the high concentration of free radicals that cause lipid peroxidation due to the induction of DOX. Similarly, Köse et al. reported that DOX administration resulted in renal cell degeneration with detectable apoptotic bodies due to ROS production [67]. Administration of H, R, and P provided renal histological treatment as normal tubules, glomeruli, and interstitial nephritis were detected. These facts are in harmony with [67,68]. The current findings point to the antioxidant involvement of H, R, and P in scavenging ROS and furthermore their anti-apoptotic and anti-inflammatory properties.

Apoptosis recreates a causative function in developing DOX toxicity in different tissues [69]. In the current work, DOX produced a significant elevation in the immunological reactivity of caspase-3 in the cytoplasm of renal cells. These results were attributed to the reactive oxidative stress-producing oxidative stress in addition to inflammation, leading to the apoptosis of tubular cells. These statements agree with Rashid et al., who attributed the activation of apoptotic DOX, which resulted in DNA injury and mitochondrial DNA damage [70]. The present work also explained the sufficient suppression of apoptosis by propolis extract in kidneys exposed to DOX. This anti-apoptotic effect of honey, royal jelly, and propolis was reported by other investigators [71]. To clarify the protective mechanisms of H, P, and R, and (H + P + R) combined at the molecular level, the expression levels of Bcl-2 and PARP-1 were evaluated. Poly (ADP-ribose) polymerase (PARP) is known to stimulate a specific response wherein the ADP-ribose moiety of NAD+ is transmitted to an amino acid receptor, producing poly (ADP-ribose) polymers. The PARP family includes 17 enzymes participating in a conserved catalytic domain [72].

Additionally, PARP-1 is mainly depicted as a key enzyme for detecting and repairing DNA damage; however, excessive activation of PARP-1 causes necrotic cell death by depleting intracellular ATP [73]. Our results indicated that treatment with DOX induces kidney injury and increases PARP-1 gene expression level two-fold. These effects were modulated by treatment with the H, P, and R, and (H + P + R) mixture. These findings were in line with other studies that point out PARP-1 inhibition of expression of adhesion molecules, infiltration in the inflammatory cells, and secondary oxidative injury in the kidney [74,75,76]. Mixed administration of royal jelly and honey diminished the cisplatin-induced alterations in diagnostic markers of both kidney and liver functions, under the effect of the capability of honey and royal jelly to scavenge free radicals, lipid peroxidation inhibition, and its anti-inflammatory roles [46,77]. Bcl-2, an anti-apoptotic gene, and its product of the Bcl-2 protein, inhibits the progression of apoptosis by the variability of oxidative stress and through interaction with mitochondrial superoxide dismutase SOD [78]. Obtained results showed that Bcl-2 gene expression was lessened in the DOX treatment group and increased significantly due to treatment with H, P, and R, and (H + P + R) combined. This effect might be due to the antioxidant potentiality of H, P, and R. Honey, propolis, and royal jelly are shown to have antioxidant properties [79,80].

## 5. Conclusions

We conclude that the administration of honey, propolis, and royal jelly significantly enhanced and resolved the renal injuries and toxicity that took place after the Doxorubicin injection. The mitigation of renal damage was in the significant improvement of kidney function assays, renal histopathology and immunohistochemistry, and mRNA of Bcl-2 and PARP-1 expression levels. Furthermore, the combined treatment significantly inhibited nephrotoxicity induced by DOX compared to the honey, propolis, and royal jelly alone. These natural products induce antioxidant effects, prevent oxidative stress, and enhance the expression level of anti-apoptotic genes.

## Data Availability

Data is contained within the article.
